# Sodium Ions Affect Pyrraline Formation in the Maillard Reaction With Lys-Containing Dipeptides and Tripeptides

**DOI:** 10.3389/fnut.2022.874650

**Published:** 2022-03-25

**Authors:** Zhili Liang, Xu Chen, Zhao Yang, Yan Liu, Xueying Qiu, Zhenzhen Zeng, Shuidi Lu, Yuehan Liu

**Affiliations:** ^1^School of Food Science, Guangdong Food and Drug Vocational College, Guangzhou, China; ^2^Engineering Research Center of Health Food Design and Nutrition Regulation, School of Chemical Engineering and Energy Technology, Dongguan University of Technology, Dongguan, China

**Keywords:** advanced glycation end products, Maillard reaction, pyrraline, salt, peptides

## Abstract

Advanced glycation end products (AGEs) are potentially-hazardous chemical compounds, produced by the Maillard reaction between reducing sugars and Lysine side-chain amino groups in proteins. AGEs are strongly associated with diabetes, Alzheimer's disease and atherosclerosis. Pyrraline, a sugar derivative of Lysine, is a major AGE and an established marker for the presence of dietary AGEs. In this study, the effects of NaCl and different dipeptide and tripeptide structures were compared on the formation of pyrraline-containing peptides and the glucose derivative 3-deoxyglucosone in the presence of glucose and at different NaCl concentrations. The physicochemical properties (polarizability, dipole moment, molecular volume and dissociation constant) and the thermodynamic properties of the peptides were determined. The amount of the pyrraline decreased significantly in the following order of peptides (at the same concentrations): Lys-Phe > Lys-Ala > Lys-Gly; Lys-Gly-Phe > Lys-Gly-Ala > Lys-Gly-Gly. The highest levels of both pyrraline and 3-deoxyglucosone occurred at 0.2 mol/L Na^+^. Sodium ions appear to alter the intramolecular electron density and charge distribution of the peptides and facilitate the reaction by stabilizing some of the intermediates in the reaction sequence.

## Introduction

The Maillard reaction (MR) is a complex series of reactions, first proposed by Louis Camille Maillard in 1912, which occurs during cooking of any food that contains both sugars and protein. It is a non-enzymatic condensation reaction between the carbonyl group of a reducing sugar and the primary amine of the Lysine side-chain in protein. The MR is very useful to the food industry because it contributes desirable brown colors, aromas and flavors during thermal food processing (1). However, thermal processing accompanied by the MR can also lead to the formation of potentially-harmful reaction products, such as advanced glycation end products (AGEs). Dietary AGEs can accumulate in the body and have a harmful effect on human health (2). Pyrraline is a major AGE, arising from a MR of the Lysine side-chain amino group, which is commonly detected in food products and is often used as an indicator of the degree of damage caused by heat treatment of foods (3). Pyrraline is easily absorbed from the human digestive system into the body (4, 5) and contributes to chronic diseases, such as diabetes, Alzheimer's disease and atherosclerosis (6, 7).

Pyrraline is commonly found in a wide range of foods. Pyrraline in milk and whey protein powders can reach 3.1 g/kg protein, and is easily transferred to processed foods made from these ingredients (8). Pyrraline can reach 3.7 g/kg protein in bakery products, especially in the outer crust of bread and cookies, 20–140 mg/kg protein in pasta (9, 10), up to 378 mg/kg protein in processed carrots and up to 382 mg/kg protein in roasted peanuts (11). The pyrraline content in processed carrots and roasted peanuts is much higher than those of other AGEs (12), indicating that pyrraline is the major AGE in many foods, and therefore, a good representation of influences on AGE formation in general.

In addition to amino acids, peptides, proteins and sugars, inorganic salts are also an important factor influencing the MR pathway. The presence of metal ions (especially Fe^2+^ and Cu^2+^) in foods accelerates the MR and increases browning, whereas the presence of high concentrations of NaCl inhibits browning (13). Addition of Ca^2+^ to an asparagine/glucose model system inhibited acrylamide formation, but promoted the production of 5-hydroxymethyl furfural and furan (14, 15). Increasing the concentration of NaCl in a wheat-dough cooking-model reduced acrylamide production (16), but NaCl only decreased acrylamide formation at concentrations of 1–2% w/w, above 2% w/v, it increased acrylamide formation (17). These findings suggest that inorganic salts are an important factor influencing the rate and mechanism of the MR, but there are no systematic studies on the influence of inorganic salts on AGE formation.

In most foods, the content of free amino acids is much lower than that of peptides and proteins (18), therefore, the content of protein-pyrraline and peptide-pyrraline conjugates in foods would be much higher than that of free pyrraline. However, the complexity of protein structure is a serious impediment to studying the effect of protein structure on pyrraline formation. After protein digestion and hydrolysis in the intestine, proteins, are absorbed in the form of free amino acids and short peptides containing 2–4 amino acid residues. Peptide-pyrraline is produced by a MR between the side chain amino group of a lysine residue in a protein or peptide and 3-deoxyglucosone (3-DG), a derivative of glucose, or glucose itself. The spatial structure and charge distribution of lysine can be influenced by adjacent amino acids (19, 20), thus affecting the rate of pyrraline formation. Therefore, this study investigated the influence of sodium ions on the formation of peptide-AGEs by the MR. The effects of different di- and tri-peptide structures on the production of their corresponding peptide-AGEs were compared at various sodium ion concentrations. The physicochemical properties (polarizability, dipole moment, molecular volume and dissociation constant) and thermodynamic properties of the peptides were calculated, to characterize the resulting peptide-AGEs.

## Materials and Methods

### Chemicals and Reagents

The three dipeptides (Lys-Gly, Lys-Ala, Lys-Phe) and three tripeptides (Lys-Gly-Gly, Lys-Gly-Ala, Lys-Gly-Phe) were from LifeTein LLC. (Beijing, China). Standards of 3-Deoxyglucosone (3-DG, purity > 99.99%) and pyrraline (purity > 99.99%) were from Toronto Research Chemicals (Toronto, ON, Canada). Formic acid (for LC-MS, 99%) was from Aladdin Biochemical Technology Co., Ltd. (Shanghai, China). Solid-phase extraction (SPE) cartridges (Cleanert PEP-2, 200 mg/6 ml) for purification were from Bonna-Agela Technologies Co., Ltd. (Tianjin, China). Chromatographic grade acetonitrile was from Merck (Darmstadt, Germany). Pepsin, pronase E, and prolidase were from Sigma-Aldrich (Shanghai, China). All other reagents were of analytical grade from Macklin Inc. (Shanghai, China).

### Glucose–Peptide–NaCl Model Systems

Six sodium chloride solutions of concentration 0.0, 0.1, 0.2, 0.3, 0.4, and 0.5 mol/L were prepared. Peptide solution (50 mM, 0.3 ml) and glucose solution (50 mM, 0.3 ml) were added to a 25-ml polytetrafluoroethylene (PTFE)-lined hydrothermal autoclave reactor (Tefic Biotech Co., Xi'an, China), then, the mixture was diluted to 15 ml with one of the sodium chloride solutions. The final concentrations of each peptide (Lys-Gly, Lys-Ala, Lys-Phe; Lys-Gly-Gly, Lys-Gly-Ala, Lys-Gly-Phe) and glucose were 1 mM. The autoclave reactor was sealed, then heated in an oil bath at 140°C for 20 min. All reactions were performed in triplicate.

After the heat treatment, the autoclave reactor was immediately cooled to room temperature in an ice/water bath, unsealed, then *o*-Phenylenediamine solution (OPD, 5 mol/L, 0.1 ml) was added to terminate the reaction, by combining with any remaining reducing sugars.

To facilitate the determination of the pyrraline content of peptides, enzymatic digestion of the peptides was performed as described previously (21). Briefly, an aliquot (~2 mg protein equivalent) of the reaction solution was removed, the pH adjusted to two and the peptide digested with pepsin solution (1 FIP-U/sample) at 37°C for 24 h. Next, the pH was adjusted to 7.5 and the peptide further digested with pronase E (400 PU/sample) at 37°C for 24 h, followed by addition of aminopeptidase (0.4 U/sample) and prolidase (1 U/sample) and the digestion continued at 37°C for 24 h. After complete enzymatic digestion, the solution was centrifuged (7,840 × g, 25 min) and the supernatant retained for solid phase extraction, as described previously (22).

### Instrumental Analysis Methods

The concentrations of pyrraline, 3-DG and lysine were determined using LC-MS in multiple ion monitoring mode, as described previously (22, 23). The pyrraline concentration was determined at M/z 255.13, 3-DG quinoxaline at M/z 235.10, and lysine at M/z 147.11. Quantitative calibration curves were constructed by comparison with an external standard mixture of pyrraline, 3-DG and lysine.

### Peptide Content Determination

Since the six peptides selected had only one lysine residue in their structures, the molar concentration of lysine in each sample after complete hydrolysis was equal to the concentration of the remaining peptide in the system after the MR.

### Calculation of Physico-Chemical and Thermodynamic Parameters

The four physicochemical parameters used to characterize the peptides were polarizability, dipole moment, volume and pK_a_. The volume was calculated by Multiwfn software, using the Marching Tetrahedron algorithm (24). The polarizability and dipole moment calculations were performed using ORCA version 4.2.1 software (25) at the B3LYP-D3/def2-TZVP level of theory, and the energy-related parameter calculations at the B3LYP/6-311++G(d,p) level in the SMD water solvent model.

For a deprotonation reaction, AH ⇋ A^−^ + H^+^, the pK_a_ was calculated as follows:


$$\begin{array}{l} \text{p}{\text{K}}_{\text{a}}\;=\;\frac{{\text{Δ}{\text{G}}^{1\text{M}}}_{\text{aq}}}{2.303\text{RT}} \end{array}$$

where *R* is the gas constant, *T* is the temperature (298.15 K), and ${\text{Δ}{\text{G}}^{1\text{M}}}_{aq}$ is the aqueous phase free energy change for the deprotonation reaction.

For the proposed reaction of peptide-sodium complex formation, peptide + Na^+^⇋ peptide~Na^+^, the change in energy after the reaction was calculated as follows:


$$\begin{array}{l} \Delta G\;=\;G_{peptide\sim Na^{+}}\;-\;\left(G_{peptide}\;+\;G_{Na^{+}}\right) \end{array}$$

### Statistical Analysis

Analysis of variance (ANOVA) of data was calculated using Origin 9.0 program (OriginLab Co., Northampton, MA, USA); comparison of means was performed by ANOVA and Duncan's multiple range test (*p* < 0.05).

## Results

### Calculated Physico-Chemical and Thermodynamic Properties of the Peptides

**Figure 1:**
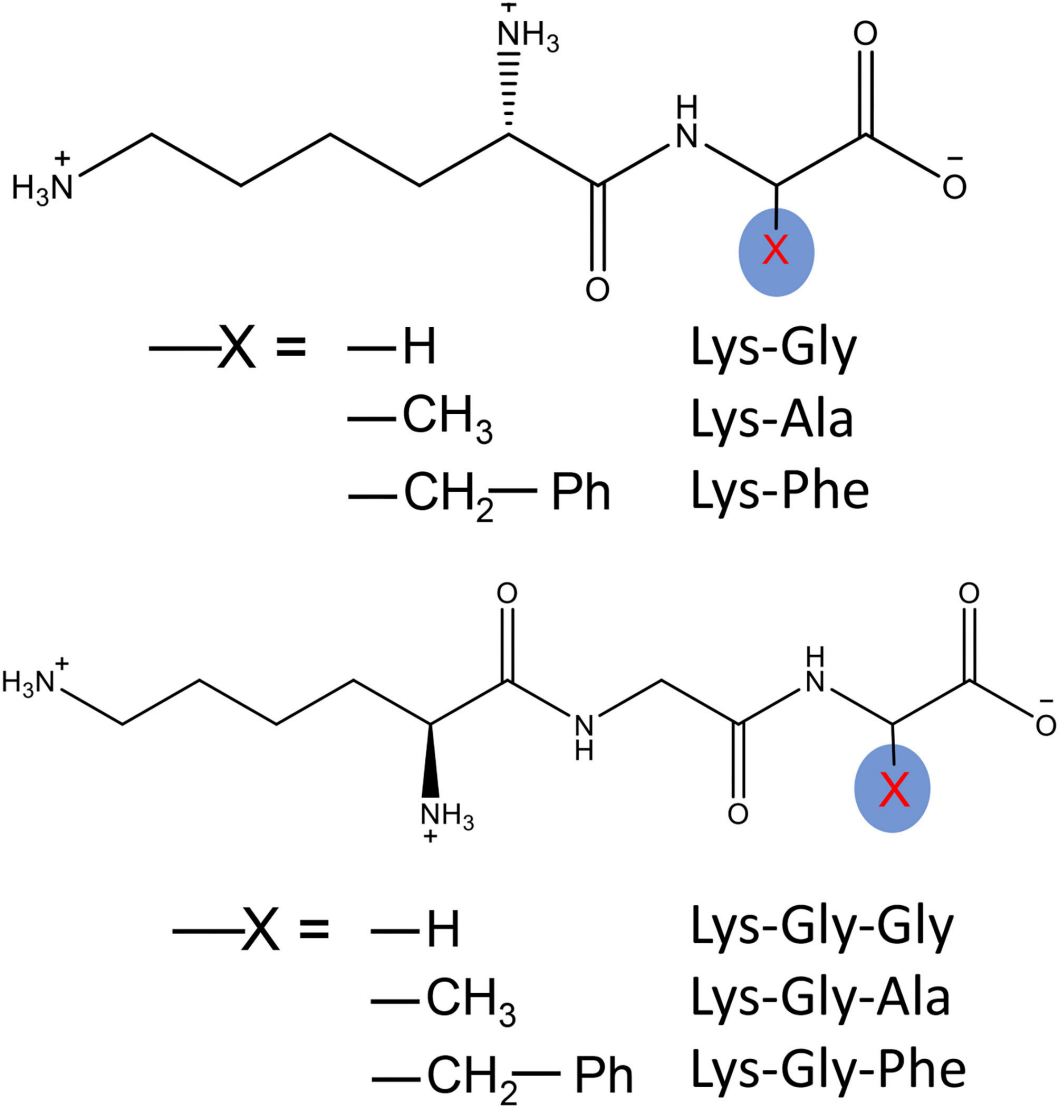
The dipeptides and tripeptides and their structures.

The peptides all had an N-terminal Lys and a C-terminal Gly, Ala, or Phe, with the tripeptides having an additional Gly in the middle position (Figure 1); these different sequences were chosen to maximize the differences in their influence on pyrraline formation during the MR. The polarizability, dipole moment and molecular volume of the three dipeptides increased in the order, Lys-Gly, Lys-Ala and Lys-Phe, as did those of the three tripeptides, Lys-Gly-Gly, Lys-Gly-Ala and Lys-Gly-Phe and the values for the tripeptides were all higher than the corresponding dipeptide (Table 1). In contrast, the pK_a_ values had the opposite trend and the tripeptides had lower values.

**Table 1:** Calculated physicochemical properties of the peptides.

**Peptide**	**Polarizability (α) (× 10^**−25**^ esu)**	**Dipole Moment** **(Debye)**	**Volume (Å^**3**^)**	**pK_**a**_**
Lys-Gly	257.89	45.90	316.57	7.64
Lys-Ala	283.46	55.42	322.70	7.29
Lys-Phe	431.95	57.59	453.34	7.26
Lys-Gly-Gly	326.49	59.43	389.94	6.92
Lys-Gly-Ala	350.68	51.57	417.16	6.91
Lys-Gly-Phe	501.96	69.72	526.29	6.90

**Figure 2:**
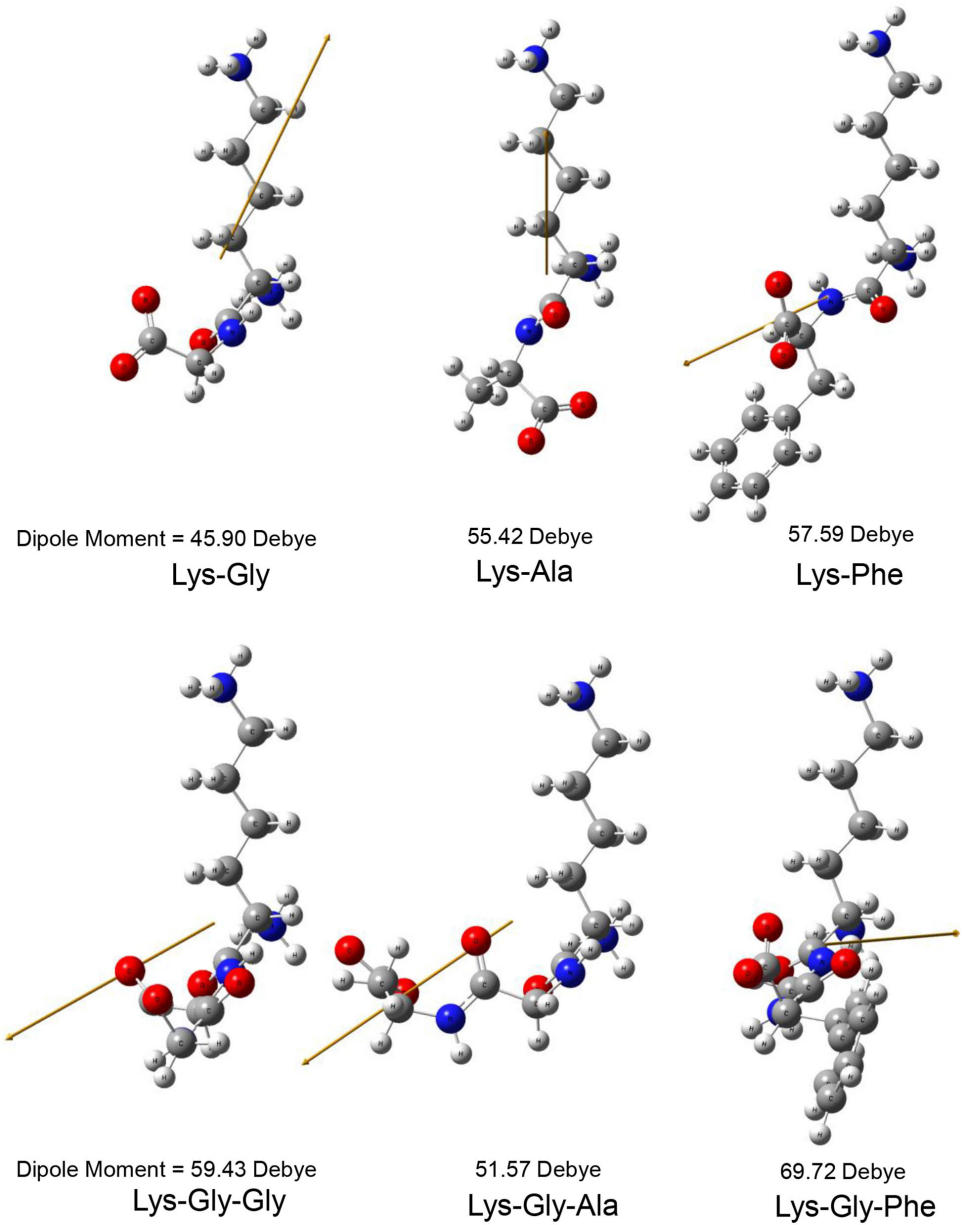
Steric structure of peptides and their dipole moments (the yellow arrow represents the dipole derivative unit vector).

**Figure 3:**
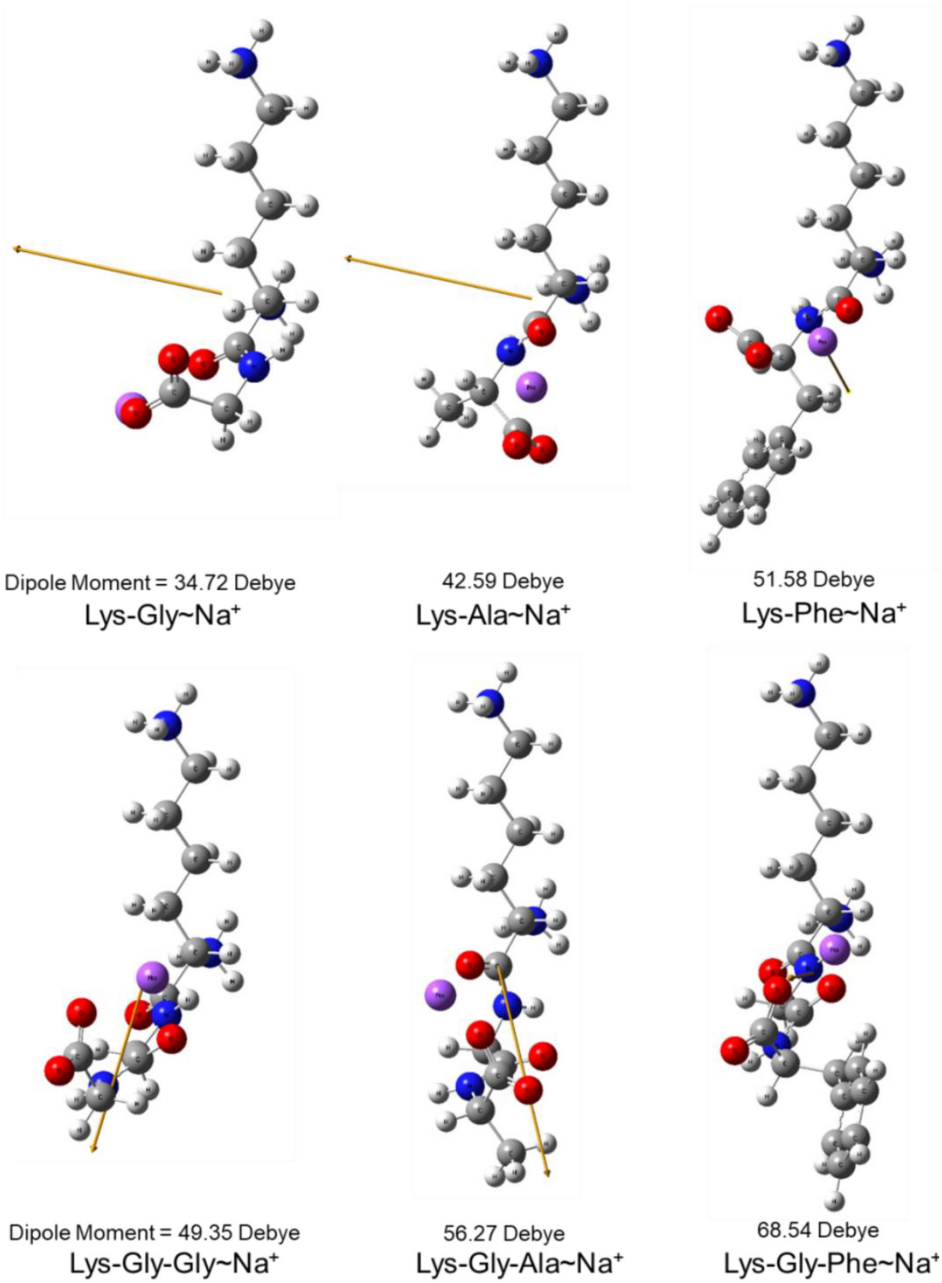
Steric structure of proposed peptide-sodium complexes and their dipole moments (the yellow arrow represents the dipole derivative unit vector and the Na^+^ ion is highlighted in purple).

The dipole moment is the product of the distance between the positive and negative charge centers and the charge carried by each charge center, so it is a vector quantity with the direction specified as pointing from the positive to the negative charge. The calculated dipole moment values and vector orientations of the peptides are shown in Figure 2 and those of the proposed peptide-sodium complexes in Figure 3.

**Figure 4:**
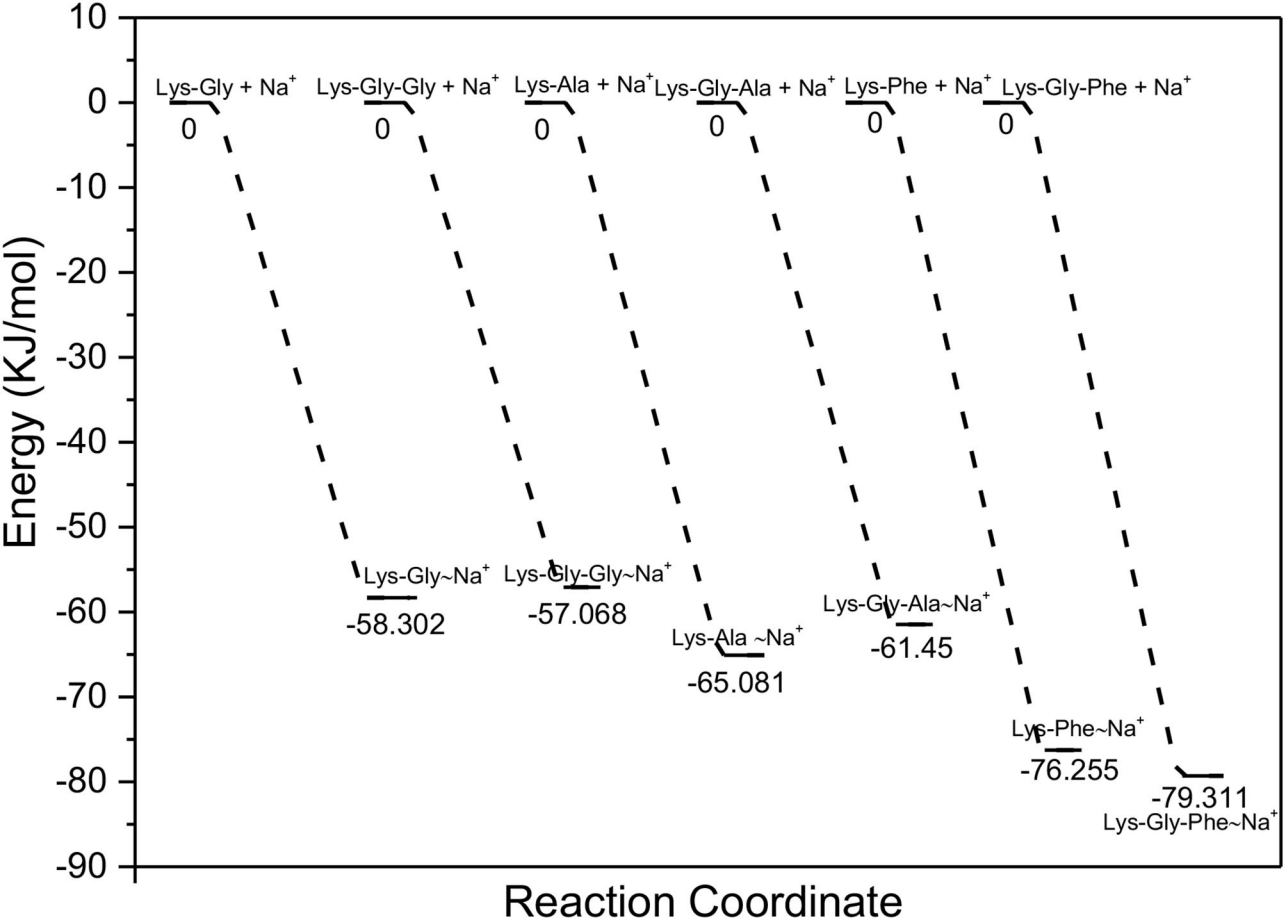
Enthalpy changes for the formation of the proposed peptide-sodium complexes.

For the proposed sodium complex formation, peptide + Na^+^ ⇋ peptide~Na^+^, the ease with which each peptide forms a transition-state, or intermediate with Na^+^ was determined by calculating the enthalpy change of the complex. The descending order of enthalpy changes was: Lys-Gly-Phe (−79.31 KJ/mol) > Lys-Gly-Ala (−61.45 KJ/mol) > Lys-Gly-Gly (−57.07 KJ/mol); Lys-Phe (−76.25 KJ/mol) > Lys-Ala (−65.08 KJ/mol) > Lys -Gly (−58.30 KJ/mol). 76.25 KJ/mol) > Lys-Ala (−65.08 KJ/mol) > Lys-Gly (−58.30 KJ/mol) (Figure 4). However, comparing each dipeptide and its corresponding tripeptide, the enthalpy changes were small and did not show a consistent trend: Lys-Gly-Gly (−57.07 KJ/mol) > Lys-Gly (−58.30 KJ/mol), Lys-Gly-Ala (−61.45 KJ/mol) < Lys-Ala (−65.08 KJ/mol), Lys-Gly-Phe (−79.31 KJ/mol) > Lys-Phe (−76.25 KJ/mol).

### Pyrraline Formation

**Figure 5:**
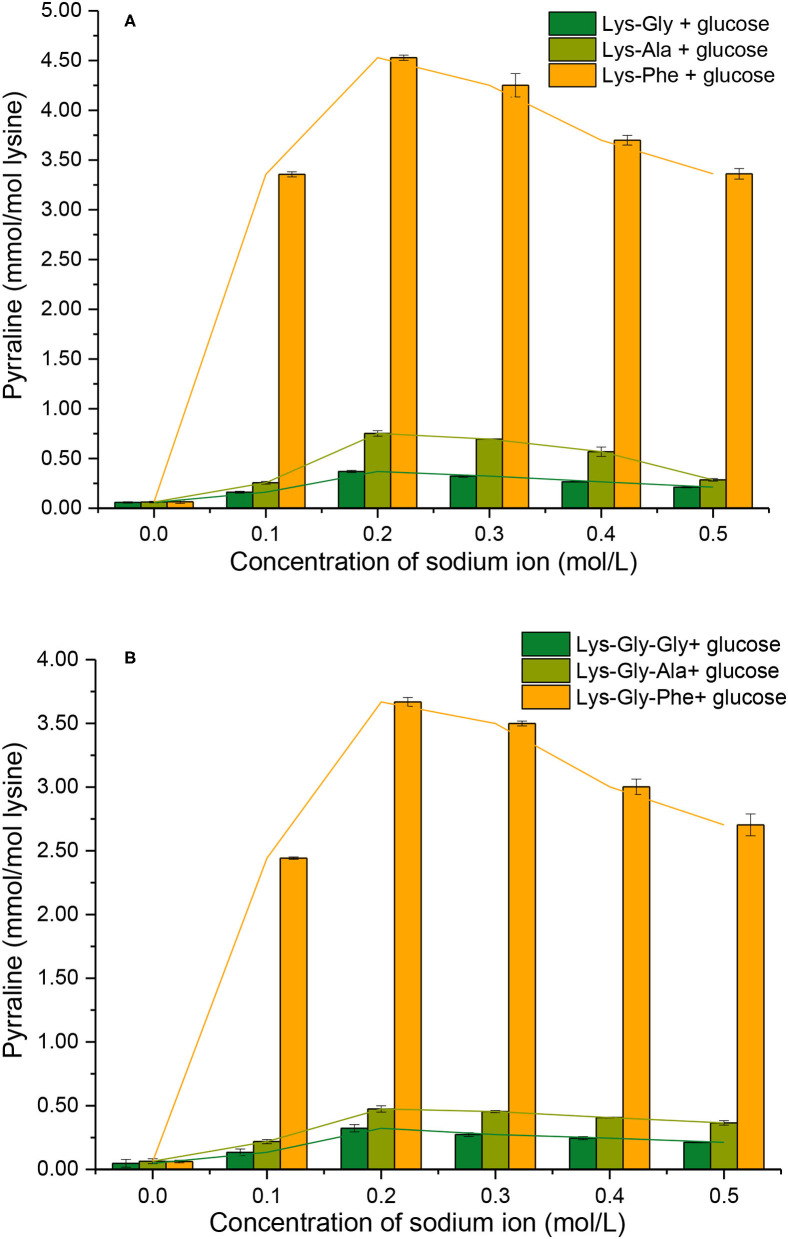
Pyrraline formation during the Maillard reaction of peptides with glucose at different sodium ion concentrations [**(A)**: in the mixtures containing dipeptides; **(B)**: in the mixtures containing tripeptides].

The extent of pyrraline formation was determined analytically after the reaction between the peptides and glucose in the presence of 0.0 to 0.5 mol/L (Figure 5). Very little pyrraline (0.060 ± 0.002 mmol/mol lysine) was produced in the absence of Na^+^, but the pyrraline content increased markedly with Na^+^ present, with a maximum at 0.2 mol/L.

Comparing the different peptides, the pyrraline content was markedly higher in the Phe-containing peptides than the others (Supplementary Table S1 in the Supplementary Information) and very significantly higher in the dipeptide reactions than the tripeptide reactions at a given Na^+^ concentration (except for 0.0 mol/L Na^+^) (Figure 5). A study of pyrazine formation during the MR found, similarly, that reaction mixtures containing dipeptides produced more pyrazine compared with those containing tripeptides (26).

The findings from this study suggest that the C-terminal amino acid of the peptide has a major influence on pyrraline formation. Although the N atom in the pyrrole ring in pyrraline is derived from the ε-NH_2_ of lysine, the reactivity of the ε-NH_2_ appears to be strongly influenced by the physicochemical properties of the adjacent amino acid residues.

### 3-Deoxyglucosone Formation

3-DG is a dicarbonyl compound derived from glucose and is thought to be an important intermediate in the formation of pyrraline and browning during the MR.

**Figure 6:**
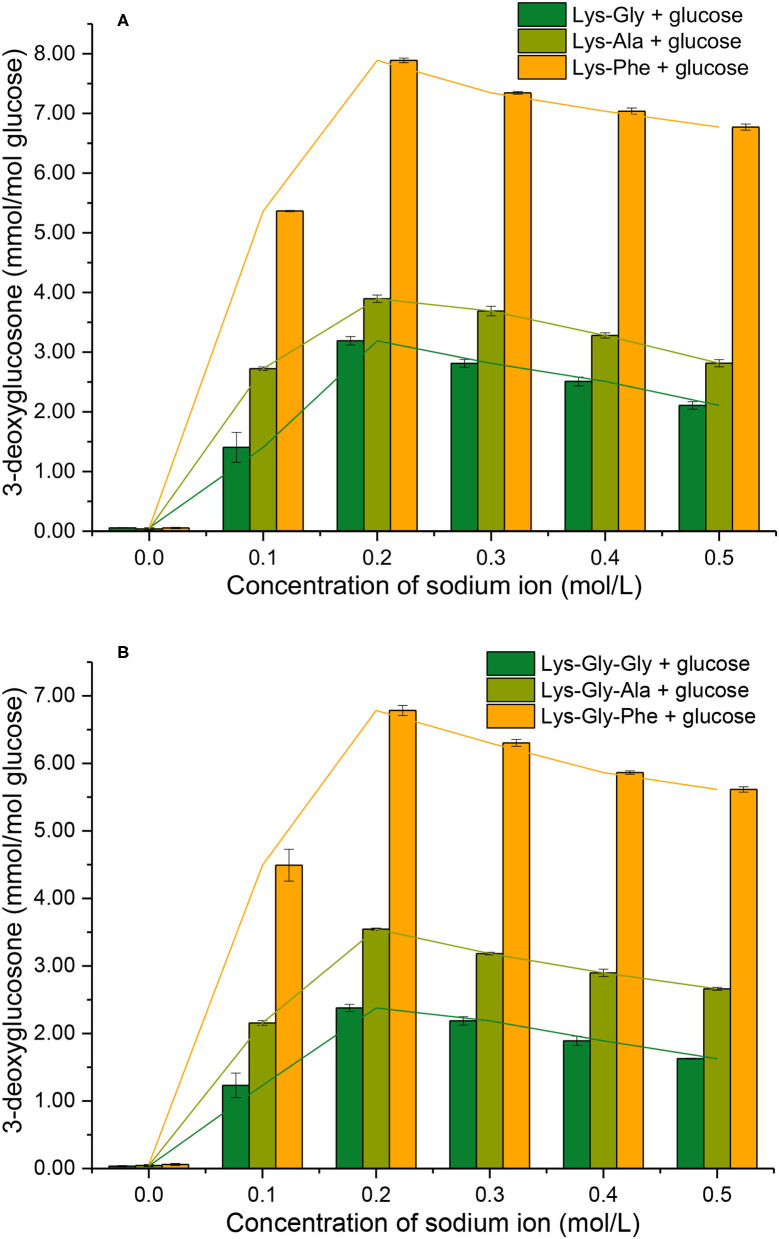
3-deoxyglucosone formation from the Maillard reaction of peptides with glucose at different sodium ion concentrations [**(A)**: in the mixtures containing dipeptides; **(B)**: in the mixtures containing tripeptides].

3-DG formation during reactions of peptides with glucose, with and without Na^+^ was determined (Figure 6). Very little 3-DG (0.060 ± 0.012 mmol/mol glucose) was produced in the absence of Na^+^, but the 3-DG content increased markedly with Na^+^ present, with a maximum at 0.2 mol/L.

Comparing the different peptides, the 3-DG content was very significantly higher in the dipeptide reactions than the tripeptide reactions, at a given Na^+^ concentration (except for 0.0 mol/L Na^+^). The 3-DG content was strongly influenced in the following order of the amino acid residues adjacent to lysine: Phe > Ala > Gly (Supplementary Table S2 in the Supplementary Information), however, the differences in 3-DG content between Phe and the others were smaller than the corresponding differences in pyrraline content (Figure 5).

There are two main mechanisms of 3-DG formation during the MR; one is the direct degradative rearrangement reaction of sugar, without involvement of amino groups (27). The other is Schiff's base formation between amino groups and glucose, followed by Amadori rearrangement to a 3-DG residue, containing a keto-amine group *via* the 1,2-enol pathway (28). However, the occurrence of sugar cleavage reactions is very limited in the absence of amine groups, so 3-DG formation by the Amadori rearrangement predominates. In the initial stages of MR, the Schiff bases are formed from an aldose and an amine group. If the activity of the amine groups involved in the MR is expressed in terms of the conversion rate of glucose involved in Schiff's base formation, the order of reactivity of glucose with various glycine polymers is: glycine dimer > glycine trimer > glycine (27).

It appears that the pathway by which glucose reacts with the peptide to form a Schiff's base and subsequently 3-DG, is influenced by the peptide structure. Thus, the amount of 3-DG formed in the various peptide-glucose mixtures may be influenced by the amino acid residues close to lysine in the peptide and their effects on the Amadori rearrangement of the Schiff base to 3-DG.

### Consumption of Peptides During the MR

**Figure 7:**
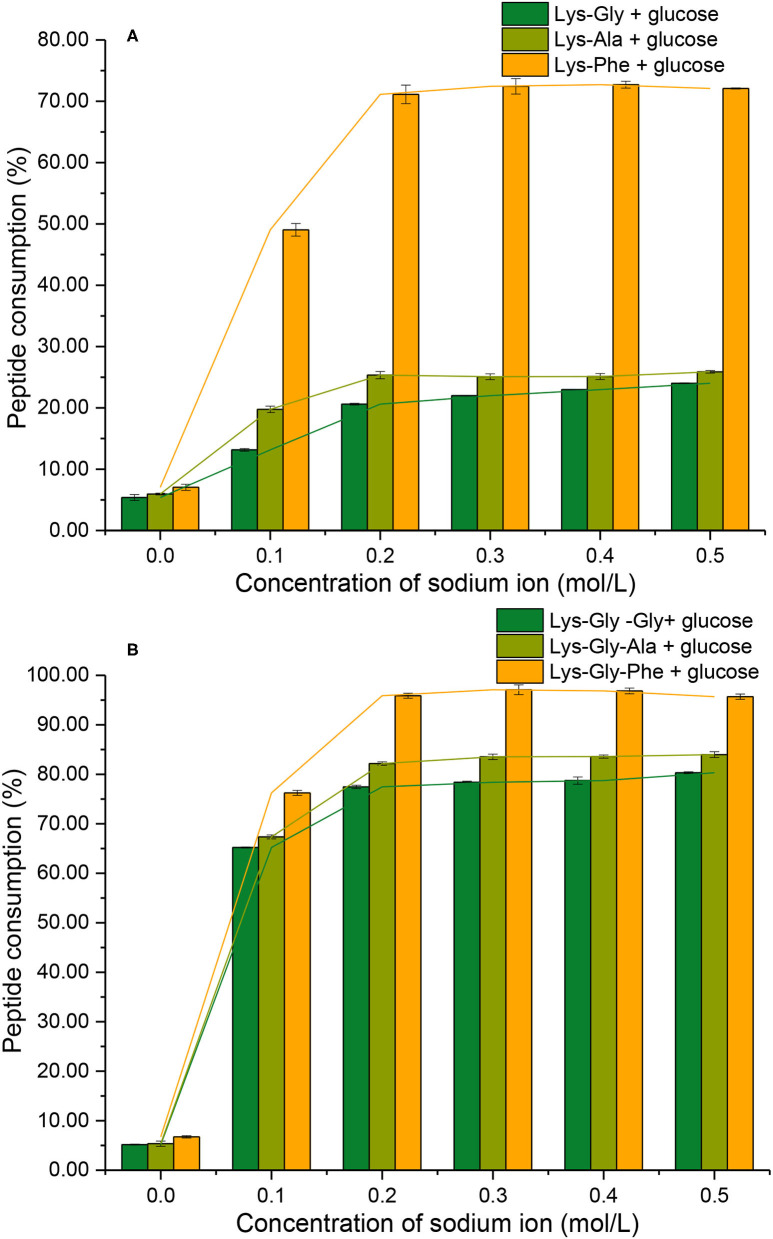
Peptide consumption after the Maillard reaction of peptides with glucose at different sodium ion concentrations [**(A)**: in the mixtures containing dipeptides; **(B)**: in the mixtures containing tripeptides].

Consumption of peptide by the MR should be related to the formation of pyrraline and 3-DG. In the absence of Na^+^, low peptide consumption was observed (mean 6.2 ± 0.5%) (Figure 7) and there was no significant difference between the six peptides. Peptide consumption increased markedly in the presence of Na^+^, reaching a maximum at 0.2 mol/L Na^+^, then plateauing at higher concentrations with all peptides. Lys-Phe consumption (~72%) was markedly higher than that of the other dipeptides (~23%), whereas Lys-Gly-Phe consumption was considerably higher (~97%), but not greatly different from that of the other tripeptides (~80%).

A comparison of peptide consumption with pyrraline-peptide formation (Figures 5A vs. 7A; Figures 5B vs. 7B) revealed a weak correlation, i.e., high consumption of non-Phe peptides resulted in relatively much lower pyrraline-peptide formation than from Phe peptides. Although the reaction between amine groups and glucose can produce pyrraline, it can also produce many other compounds by aldol-amine condensation at the later stages of the MR and the diversity and structural complexity of these other MR products (29) makes their quantification, relative to pyrraline, impractical.

## Discussion

### Effect of Peptide Structures on the Maillard Reaction

Chemically, pyrraline is the 2-formyl-5-(hydroxymethyl)pyrrole derivative of lysine. It is generally formed in the late stages of the MR by the reaction of the ε-NH_2_ amino group of lysine with glucose, followed by structural rearrangement, or 1, 2-dicarbonyl compounds generated by structural rearrangement of glucose, in particular by the reaction with 3-DG (21, 30, 31).

In summary, the formation of peptide-pyrralines was mainly influenced by and correlated with the polarizability of the starting peptide (Figure 5 and Table 1). There is a positive correlation between the polarizability and the flexibility of the peptide (32–35). In addition, polarizability is an important quantitative indicator of the tendency of nucleophilic reagents to participate in reactions; nucleophilic reactions usually follow the “decreasing polarizability” rule, i.e., they tend to proceed in the direction of decreasing polarizability (36).

During the early stages of the MR, the carbonyl group of the reducing sugar undergoes a condensation reaction with the nucleophilic ε-NH_2_ amino group of Lys, to form a Schiff's base, followed by an Amadori rearrangement to form a keto-amine. Therefore, the polarizability of the amino acid close to the lysine has a strong influence on the rate of Schiff's base formation because it influences the nucleophilicity of the Lysine ε-NH_2_ amino group (37–39). According to Pearson's hard and soft acids and bases (HSAB) principle (40), peptides with high polarizability (e.g., Lys-Phe and Lys-Gly-Phe) have higher reactivity with polarizable substrates (e.g., carbonyl groups of glucose), whereas peptides with low polarizability (e.g., Lys-Gly, Lys-Gly-Gly) are less reactive. Polarizability also correlated with the ease of formation of the peptide-sodium complexes (Figure 3). Therefore, Schiff's base formation and rearrangement to produce 3-DG during the early stages of the MR are accelerated by increased peptide polarizability. In addition, the reaction of 3-DG with a peptide to form peptide-pyrralines, during the later stages of the MR is similarly accelerated, because differences in peptide structure affect the electron cloud density and distribution of the whole peptide molecule.

There was a strong positive correlation between the size of the C-terminal amino acid (Phe≫ Ala > Gly) and the molecular volume of the peptide (Figure 1 and Table 1). Molecular volume is an indicator of potential steric hindrance, or blockage of a reactive center, which can result in a reduction in its reactivity (41). Although both the Lysine α-NH_2_ and ε-NH_2_ can be involved in the MR, and the reactivity of the α-NH_2_ is higher than that of ε-NH_2_ (31), the pyrraline structure can only be derived from the reaction of 3-DG with the ε-NH_2_. In Lys-Phe and Lys-Gly-Phe, the large volume of the phenyl side-chain sterically hinders the reactivity of the α-NH_2_ of lysine, whereas the more distant ε-NH_2_ is much less affected by steric hindrance. The probability of α-NH_2_ being involved in the MR is reduced, while the probability of ε-NH_2_ is not affected. There is an increased probability that the MR will proceed along the pyrraline formation pathway. Therefore, the Phe-containing dipeptides and tripeptides are more likely to form peptide-pyrralines. In addition, the peptide may undergo degradation under high temperature conditions, but the large Phe side chain in Lys-Phe and Lys-Gly-Phe help to stabilize the peptide by cation-π interaction (42). It appears that peptides with Phe side chain are more conducive to peptide-pyrraline formation.

The acid dissociation constant represents the ability of a substance to dissociate from hydrogen ions or protons. For the deprotonation reaction AH ⇋ A^−^ + H^+^, the pK_a_ value is reduced when the interaction of molecules is biased toward the deprotonated state (A^−^) ([A^−^] to [AH] ratio increases). Conversely, the pK_a_ value is raised when the molecular interaction is biased toward the protonated state (AH). For the three dipeptides and tripeptides, the order of their pK_a_ values is: Lys-Gly > Lys-Ala > Lys-Phe≫ Lys-Gly-Gly > Lys-Gly-Ala > Lys-Gly-Phe. This order also represents their ability to be deprotonated, i.e., the probability of presenting a deprotonated state under the same conditions. In neutral solutions, the α-NH_2_ and ε-NH_2_ on the lysine residues in the various peptide structures are in the positive ionic form. They are present in non-positive ionic form only after a deprotonation reaction has occurred. Therefore, peptides with high pK_a_ values under the same conditions also have a higher number of non-ionic N in their structure. Yamaguchi's findings show that the reaction of amino acids with glucose is difficult to occur when the amino acid residues are in positive ionic form (43). The reaction in the early stages of the MR is the nucleophilic addition of N to the carbonyl group, which must be in the non-ionic form (i.e., protonated state) at the N terminus. The rate of the amino-carbonyl reaction in the MR depends on the lone pair of electrons, whereas non-ionic amino acids have a lone pair of electrons on N, which is more conducive to the MR. These are also important factors in explaining the formation of pyrraline (Figure 5) and 3-DG (Figure 6).

Differences in hydrophobicity may also contribute to the differences in pyrraline and 3-DG formation between the different peptides. Hydrophobic interactions between apolar molecules or apolar parts of molecules in aqueous solutions can accelerate organic chemical reactions, probably because they destabilize the initial state of the reaction and promote the formation of the reaction transition state, which accelerates the reaction (44).

As is apparent from Figure 1, the hydrophobicity of the peptides is in the following order: Lys-Phe > Lys-Ala > Lys-Gly; Lys-Gly-Phe > Lys-Gly-Ala > Lys-Gly-Gly. Peptides containing Ala and Phe, especially those containing Phe, produced more peptide-pyrraline, therefore, peptides with higher hydrophobicity may contribute to enhanced peptide-pyrraline formation.

### Effect of NaCl Concentration on the Reaction

In this study, pyrraline and 3-DG formation, as well as peptide consumption, were markedly accelerated in the presence of Na^+^ ions, with the greatest acceleration at 0.2 mol/L Na^+^. Higher Na^+^ concentrations produced no further increase, or a small decrease. Nucleophilic and electrophilic organic reactions are influenced by changes in intramolecular electron density distribution and the main mode of action of metal ion catalysts is to initiate such electron density changes. Metal ions induce major electron density changes when coordinated with organic substrates, so that reactions dependent on electron rearrangement may be promoted by metal ions (45). Even in concerted reactions, metal ions can perturb the rate coefficients by more than an order of magnitude, through interactions with charge-separated transition states (46–49) and may accelerate, or inhibit the reaction. The rate of most of reaction steps of glucose pyrolysis increased, which is consistent with the experimental observation that the rate of reaction increases in the presence of inorganic salts (50, 51). The reason why high concentrations of NaCl (> 0.2 mol/L) in this study reduced pyrraline and 3-DG formation is not clear. The effectiveness of metal ion catalysis depends not only on the type of reaction, but also on the stereochemistry in and around the reaction center (52). The electric dipole moment measures the separation of the positive and negative charge distribution in the molecular structure, that is, the overall polarity of the molecule and the orientation of the dipole moment is from negative to positive charge. When an Na^+^ ion interacted with the peptide structures, the calculated dipole moment values for each peptide changed to varying degrees, and the orientation of the dipole moment also changed markedly (compare Figures 2, 3), i.e., the presence of Na^+^ markedly altered the charge distributions of the peptides. In addition, there is a greater extent of perturbation of the electronic structure of α-glucose by Na^+^ than that of β-glucose. The effect of inorganic salts on glucose cleavage products may cause a shift in the equilibrium ratio of glucose between α- and β-glucose isomers or cause a selective redistribution of electron density in the reaction process (53).

Many reactions require the presence of an “electron sink” in the molecule to absorb the electron density generated by the reaction transition state, or an intermediate species. The reaction between glucose/3-DG and an amino group requires neutralization of the negative charge to reduce electrostatic repulsion (45) and the addition of metal ions may serve this purpose.

It is possible for metal ions to interact electrostatically with both components of a bimolecular reaction, thereby holding them in close proximity and facilitating transition state formation, and may also assist the reaction through an increase in entropy. In addition, nucleophilic substitutions will be accelerated by the presence of a metal ion if both molecules contain a whole or partial negative charge because the electrostatic repulsion between the molecules is reduced. Metal ions can also facilitate the reaction by stabilizing a transition state, or intermediate. In the reaction between glucose/3-DG and an amino group, the Na^+^ ion does not interact with the ground state, but may facilitate the formation and stabilization of an intermediate or a transition state in the reaction sequence (45). This effect, of stabilizing a reaction intermediate or a transition state, is consistent with the negative enthalpy changes calculated for binding of sodium to the peptides (Figure 4).

## Conclusion

This study aimed to increase knowledge of the mechanism of the Maillard reaction (MR), between protein (Lysine) amino groups and reducing sugars, which can produce potentially toxic advanced glycation end products (AGE), such as pyrraline. Comparison of pyrraline and 3-deoxyglucosone (3-DG) yields from different model peptides, in the presence of glucose and various concentrations of NaCl, showed that a highly polarizable amino acid residue (e.g., Phe) close to the reactive Lys residue strongly accelerated the reactions that produce pyrraline and 3-DG, compared with less-polarizable Ala or Gly. However, the dominant influence on these reactions was the presence of NaCl, i.e., sodium ions, which strongly catalyzed the reaction; in the absence of NaCl, there was negligible pyrraline, or 3-DG formed from any model peptide reaction.

The MR mainly occurs during cooking, or other heat treatment of food products containing both sugar and protein, during industrial processing and initially produces both an attractive brown color and desirable flavors, but can also produce undesirable AGEs after extended heating. The findings of this study have two implications for minimizing AGE formation during food processing, thereby minimizing the content of potential toxins. The main one is that the salt concentration in the food should be minimized as much as possible before heat treatment and any salt in the final formulation should be added later. In addition, where practical, proteins with amino acid sequences containing multiple Lys residues, close to a polarizable residue such as Phe, should not be heated in the presence of sugar. These findings may have great utility to the food industry and form the basis for further studies on minimizing AGE formation in processed foods.

## Data Availability Statement

The original contributions presented in the study are included in the article/Supplementary Material, further inquiries can be directed to the corresponding author/s.

## Author Contributions

ZL: funding acquisition, investigation, methodology, writing—original draft, and writing—review and editing. XC: conceptualization, funding acquisition, and methodology. ZY: writing—review and editing. XQ, ZZ, SL, YaL, and YuL: investigation and methodology. All authors contributed to manuscript revision, read, and approved the submitted version.

## Funding

This work was financially supported by the National Natural Science Foundation of China (No. 31801667), Natural Science Foundation of Guangdong Province (No. 2020A1515011341), Dongguan Social Science and Technology Development Key Project in 2021 (20211800904672), the Foundation for Innovation Team in Higher Education of Guangdong, China (No. 2021KCXTD035), and the Institute of Science and Technology Innovation of DGUT, China (KCYCXPT2017007).

## Conflict of Interest

The authors declare that the research was conducted in the absence of any commercial or financial relationships that could be construed as a potential conflict of interest.

## Publisher's Note

All claims expressed in this article are solely those of the authors and do not necessarily represent those of their affiliated organizations, or those of the publisher, the editors and the reviewers. Any product that may be evaluated in this article, or claim that may be made by its manufacturer, is not guaranteed or endorsed by the publisher.
