# Supplementary material for: Sodium Ions Affect Pyrraline Formation in the Maillard Reaction With Lys-Containing Dipeptides and Tripeptides
Source: Front Nutr. 2022 Mar 25;9:874650. doi: 10.3389/fnut.2022.874650 (PMC8990318; doi:10.3389/fnut.2022.874650)
Supplement: Supplementary file 1 [file Table_1.DOCX]

**Supplementary Information for**

Sodium Ions Affect Pyrraline Formation in the Maillard Reaction with Lys-Containing Dipeptides and Tripeptides

Zhili Liang ^1,*^, Xu Chen ^2^, Zhao Yang ^1^, Yan Liu ^1^, Xueying Qiu ^1^, Zhenzhen Zeng ^1^, Shuidi Lu ^1^, Yuehan Liu ^1^

^1^ School of Food Science, Guangdong Food and Drug Vocational College, Guangzhou 510520, China

^2^ Engineering Research Center of Health Food Design & Nutrition Regulation, School of Chemical Engineering and Energy Technology, Dongguan University of Technology, Dongguan 523808, China

* Correspondence: liangzl@gdyzy.edu.cn; Tel.: +86-20-29164643

Table S1. Pyrraline formation from different peptides with glucose at different sodium ion concentrations

| Concentration of sodium ion in the mixtures (mol/L) | Pyrraline concentration (mmol/mol lysine) | | | | | |
| --- | --- | --- | --- | --- | --- | --- |
|  | Lys-Gly+glucose | Lys-Ala+glucose | Lys-Phe+glucose | Lys-Gly-Gly+glucose | Lys-Gly-Ala+glucose | Lys-Gly-Phe+glucose |
| 0.0 | 0.058 ± 0.007 ^aa^ | 0.063 ± 0.008 ^aa^ | 0.065 ± 0.016 ^aa^ | 0.058 ± 0.030 ^aa^ | 0.064 ± 0.019 ^aa^ | 0.062 ± 0.010 ^aa^ |
| 0.1 | 0.161 ± 0.009 ^b^ | 0.256 ± 0.014 ^ab^ | 3.357 ± 0.026 ^bb^ | 0.134 ± 0.026 ^cb^ | 0.218 ± 0.017 ^db^ | 2.442 ± 0.010 ^eb^ |
| 0.2 | 0.369 ± 0.012 ^c^ | 0.752 ± 0.028 ^ac^ | 4.529 ± 0.027 ^bc^ | 0.323 ± 0.029 ^cc^ | 0.475 ± 0.025 ^dc^ | 3.668 ± 0.035 ^ec^ |
| 0.3 | 0.323 ± 0.012 ^d^ | 0.695 ± 0.001 ^ad^ | 4.252 ± 0.117 ^bd^ | 0.273 ± 0.015 ^cd^ | 0.454 ± 0.008 ^dd^ | 3.499 ± 0.019 ^ed^ |
| 0.4 | 0.265 ± 0.004 ^e^ | 0.568 ± 0.047 ^ae^ | 3.699 ± 0.048 ^be^ | 0.245 ± 0.013 ^ce^ | 0.406 ± 0.004 ^de^ | 3.002 ± 0.061 ^ee^ |
| 0.5 | 0.212 ± 0.006 ^f^ | 0.285 ± 0.013 ^af^ | 3.361 ± 0.053 ^bf^ | 0.212 ± 0.004 ^cf^ | 0.365 ± 0.017 ^df^ | 2.703 ± 0.086 ^ef^ |

Those means in the same vertical column or horizontal row marked with the same superscript letter are not significantly different at the 0.05 level.

Table S2. 3-deoxyglucosone formation in the Maillard reaction between different peptides and glucose at different sodium ion concentrations

| Concentration of sodium ion in the mixtures (mol/L) | 3-deoxyglucosone concentration (mmol/mol glucose) | | | | | |
| --- | --- | --- | --- | --- | --- | --- |
|  | Lys-Gly+glucose | Lys-Ala+glucose | Lys-Phe+glucose | Lys-Gly-Gly+glucose | Lys-Gly-Ala+glucose | Lys-Gly-Phe+glucose |
| 0.0 | 0.055 ± 0.006 ^aa^ | 0.038 ± 0.021 ^aa^ | 0.055 ± 0.011 ^aa^ | 0.035 ± 0.010 ^aa^ | 0.045 ± 0.015 ^aa^ | 0.060 ± 0.016 ^aa^ |
| 0.1 | 1.405 ± 0.251 ^b^ | 2.723 ± 0.028 ^ab^ | 5.365 ± 0.010 ^bb^ | 1.232 ± 0.181 ^cb^ | 2.155 ± 0.035 ^db^ | 4.492 ± 0.236 ^eb^ |
| 0.2 | 3.189 ± 0.072 ^c^ | 3.895 ± 0.063 ^ac^ | 7.889 ± 0.039 ^bc^ | 2.378 ± 0.054 ^cc^ | 3.546 ± 0.016 ^dc^ | 6.784 ± 0.075 ^ec^ |
| 0.3 | 2.812 ± 0.066 ^d^ | 3.689 ± 0.079 ^ad^ | 7.344 ± 0.022 ^bd^ | 2.187 ± 0.063 ^cd^ | 3.183 ± 0.019 ^dd^ | 6.303 ± 0.050 ^ed^ |
| 0.4 | 2.510 ± 0.074 ^e^ | 3.280 ± 0.045 ^ae^ | 7.037 ± 0.054 ^be^ | 1.891 ± 0.068 ^ce^ | 2.898 ± 0.055 ^de^ | 5.864 ± 0.025 ^ee^ |
| 0.5 | 2.107 ± 0.063 ^f^ | 2.815 ± 0.061 ^af^ | 6.771 ± 0.051 ^bf^ | 1.627 ± 0.006 ^cf^ | 2.660 ± 0.022 ^df^ | 5.614 ± 0.040 ^ef^ |

Those means in the same vertical column or horizontal row marked with the same superscript letter are not significantly different at the 0.05 level.

Table S3. Peptide consumption in the Maillard reaction between different peptides and glucose at different sodium ion concentrations

| Concentration of sodium ion in the mixtures (mol/L) | Peptide loss (%) | | | | | |
| --- | --- | --- | --- | --- | --- | --- |
|  | Lys-Gly+glucose | Lys-Ala+glucose | Lys-Phe+glucose | Lys-Gly-Gly+glucose | Lys-Gly-Ala+glucose | Lys-Gly-Phe+glucose |
| 0.0 | 5.38 ± 0.49 ^aa^ | 5.95 ± 0.15 ^aa^ | 6.05 ± 0.50 ^aa^ | 5.87 ± 0.59 ^aa^ | 5.36 ± 0.52 ^aa^ | 6.74 ± 0.24 ^ea^ |
| 0.1 | 13.15 ± 0.22 ^b^ | 19.76 ± 0.52 ^ab^ | 49.04 ± 1.03 ^bb^ | 65.21 ± 0.10 ^cb^ | 67.36 ± 0.40 ^db^ | 76.24 ± 0.50 ^eb^ |
| 0.2 | 20.61 ± 0.35 ^c^ | 25.35 ± 0.60 ^ac^ | 71.13 ± 1.52 ^bc^ | 77.46 ± 0.35 ^cc^ | 82.17 ± 0.35 ^dc^ | 95.89 ± 0.50 ^ec^ |
| 0.3 | 21.99 ± 0.43 ^c^ | 25.09 ± 0.47 ^ac^ | 72.44 ± 1.26 ^bc^ | 77.39 ± 0.22 ^cc^ | 82.53 ± 1.54 ^dc^ | 97.08 ± 0.99 ^ed^ |
| 0.4 | 22.98 ± 0.71 ^c^ | 25.12 ± 0.49 ^ac^ | 72.71 ± 1.56 ^bc^ | 78.73 ± 0.73 ^cc^ | 82.58 ± 0.33 ^dc^ | 96.85 ± 0.59 ^ed^ |
| 0.5 | 23.01 ± 0.68 ^c^ | 25.58 ± 0.20 ^ac^ | 72.09 ± 0.10 ^bc^ | 79.32 ± 0.79 ^cc^ | 82.98 ± 0.59 ^dc^ | 96.69 ± 0.73 ^ed^ |

Those means in the same vertical column or horizontal row marked with the same superscript letter are not significantly different at the 0.05 level.
